# Circulating micronutrient levels and respiratory infection susceptibility and severity: a bidirectional Mendelian randomization analysis

**DOI:** 10.3389/fnut.2024.1373179

**Published:** 2024-08-08

**Authors:** Zhengxiao Wei, Qingqing Xiong, Li Liang, Zhangjun Wu, Zhu Chen

**Affiliations:** ^1^Department of Clinical Laboratory, Public Health Clinical Center of Chengdu, Chengdu, Sichuan, China; ^2^Department of Scientific Research and Teaching, Public Health Clinical Center of Chengdu, Chengdu, Sichuan, China; ^3^Department of Tuberculosis, Public Health Clinical Center of Chengdu, Chengdu, Sichuan, China

**Keywords:** micronutrients, Mendelian randomization, upper respiratory tract infection, susceptibility, copper

## Abstract

**Background:**

Limited and inconclusive data from observational studies and randomized controlled trials exist on the levels of circulating micronutrients in the blood and their association with respiratory infections.

**Methods:**

A Mendelian randomization (MR) analysis was conducted to assess the impact of 12 micronutrients on the risk of three types of infections [upper respiratory tract infections (URTI), lower respiratory tract infections (LRTI), and pneumonia] and their 14 subtypes. This study utilized a bidirectional MR approach to evaluate causal relationships and included a range of sensitivity analyses and multivariable MR to address potential heterogeneity and pleiotropy. The threshold for statistical significance was set at *p* < 1.39 × 10^−3^.

**Results:**

Meta-analysis revealed that higher levels of circulating copper were significantly associated with a reduced risk of URTI (odds ratio (OR) = 0.926, 95% CI: 0.890 to 0.964, *p =* 0.000195). Additionally, copper demonstrated a suggestive association with a reduced risk of LRTI (*p =* 0.0196), and Vitamin B6 was nominally associated with a reduced risk of pneumonia (*p =* 0.048). Subtype analyses further indicated several suggestive associations: copper reduces the risk of acute pharyngitis (*p =* 0.029), vitamin C increases the risk of critical care admissions for pneumonia (*p =* 0.032) and LRTI (*p =* 0.021), and folate reduces the risk of viral pneumonia (*p =* 0.042). No significant connections were observed for other micronutrients.

**Conclusion:**

We observed a genetically predicted potential protective effect of copper in susceptibility to upper respiratory infections. This provides new insights for further research into the role of micronutrients in the prevention and treatment of infection.

## Introduction

Infections have long been considered one of the major causes of significant health loss globally. Specifically, respiratory infections are a leading cause of morbidity and mortality annually worldwide (1). Acute upper respiratory tract infections (URTI) are among the most common diagnoses in global primary care due to their high incidence rate, with 17.2 billion cases occurring annually worldwide (2). Lower respiratory tract infections (LRTI) represent the primary infectious factor contributing to mortality and rank as the fifth-leading cause of death globally (3). LRTI was a major cause of illness and death in children, particularly those under five years of age (3). Pneumonia, the most common type of LRTI, accounts for 30% of all respiratory system deaths, according to data from the Organization for Economic Cooperation and Development (4). Infections impose a heavy burden on families and societies. Thus, preventing, diagnosing early, and treating these infections pose significant challenges to public health systems, making it crucial to identify modifiable risk factors for these infections. Today, while some infection-related risk factors, such as BMI (5) and lifetime smoking (6), have been identified, the role of circulating micronutrients in the pathogenesis of infections remains unclear.

Micronutrients in the blood, including various vitamins (such as vitamin A, vitamin B6, vitamin B12, folate, vitamin C, vitamin D, and vitamin E) and trace elements [like copper (Cu), zinc (Zn), selenium (Se), magnesium (Mg)], play a crucial role in the immune system (7). Therefore, their deficiency can increase the likelihood of host infections. Conversely, severe or recurrent infections can also increase the risk of malnutrition. Observational studies have found that deficiencies in vitamins A and C are associated with increased susceptibility to respiratory infections (8). However, some randomized controlled trials have produced inconsistent results, indicating that supplementation with vitamins A and C does not improve the incidence or duration of LRTI (9, 10). Other researchers believe that lower levels of vitamin D are associated with an increased risk and severity of acute respiratory infections (11, 12), yet some studies show contrary evidence, finding no difference in vitamin D status between LRTI patients and control groups (13), and no significant correlation between low levels of vitamin D and a higher rate of influenza (14). It is noteworthy that these observational study results vary greatly, potentially influenced by underlying confounding factors or reverse causality, which is unavoidable in traditional epidemiological research.

Mendelian randomization (MR), utilizing genetic variations as instrumental variables (IVs), effectively circumvents the confounding factors often unmanageable in observational studies and minimizes the likelihood of reverse causation. This approach is widely used to assess causal relationships between risk factors and diseases. In the absence of randomized controlled trials (RCTs) or when initiating new RCTs is not feasible, MR serves as a crucial alternative strategy, offering reliable evidence on the causal links between exposures and disease risks (15).

Given the lack of evidence on the relationship between circulating micronutrients in blood and respiratory infections, we aimed to integrate genome-wide association study (GWAS) resources to evaluate their causal links using the MR approach. We hypothesized that levels of micronutrients are associated with the risk of respiratory tract infections. Specifically, we identified 12 micronutrients [calcium (Ca), beta-carotene, Cu, folate, Fe, Mg, phosphorus, Se, vitamin B6, vitamin C, vitamin D, and Zn] related to infection and assessed their relationship with URTI, LRTI, pneumonia, and their 14 common subtypes, providing feasible strategies for early prevention and improvement of respiratory infection.

## Materials and methods

### Study design

**Figure 1:**
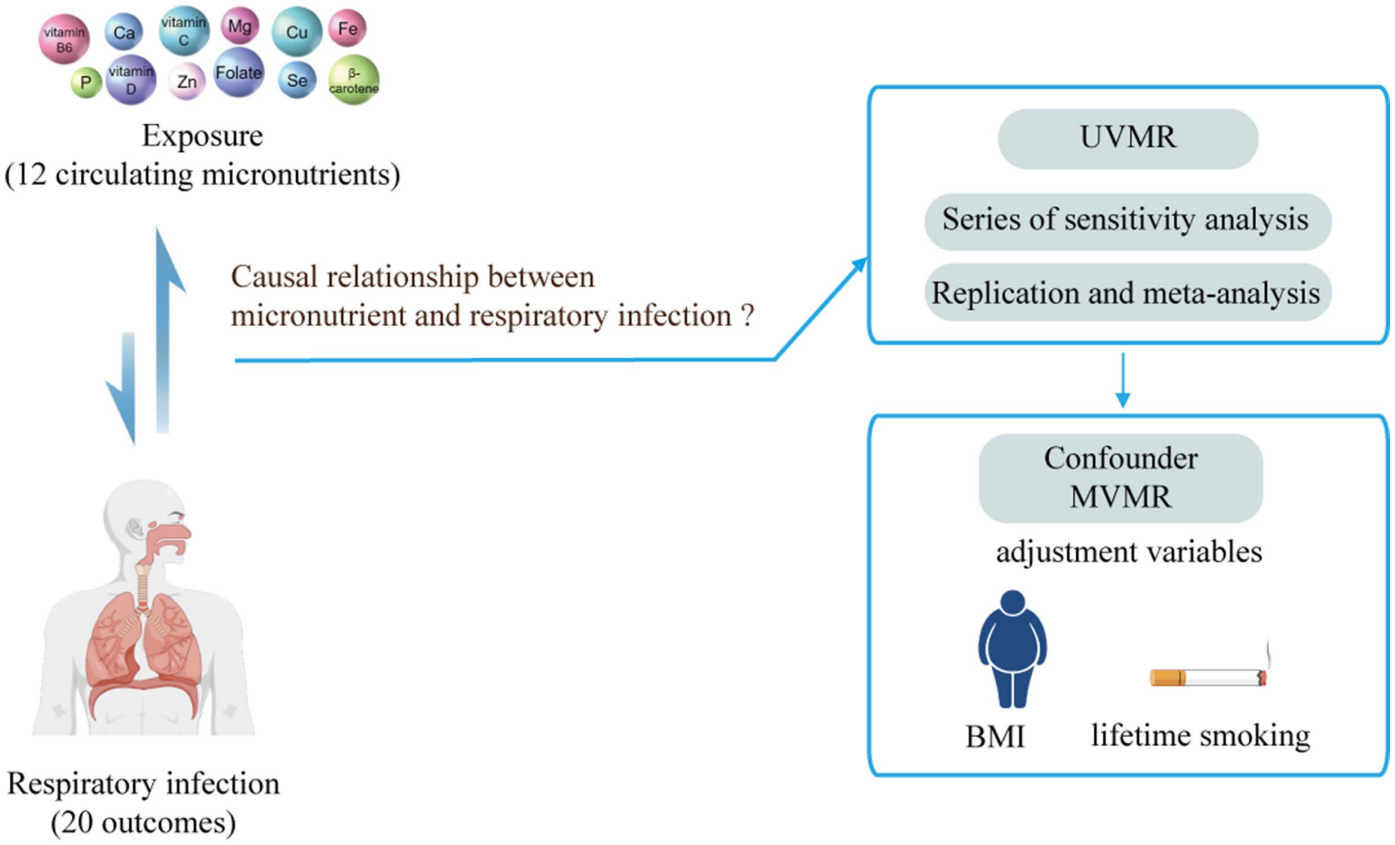
Using Mendelian randomization to study the causal relationship between micronutrients and respiratory infection. UVMR, Univariate Mendelian randomization; MVMR, Multivariate Mendelian randomization; BMI, body mass index.

This study is reported according to the STROBE-MR guidelines (Supplementary Table S1). Figure 1 and Supplementary Figure S1 illustrate the study design, outlining the included studies and key steps. Our MR analysis adhered to three assumptions. Firstly, the selected instrumental variables (IVs) must be strongly associated with the micronutrients. Secondly, the IVs should not be linked with confounding factors that affect both micronutrients and respiratory infections. Thirdly, the instrumental variables should influence respiratory infections only through the micronutrients, thus avoiding horizontal pleiotropy.

In summary, we conducted a comprehensive MR study using publicly available summary data from 32 GWAS studies, to assess the relationship between micronutrient levels and various respiratory infections. Our analysis included data from 12 exposure and 20 outcome studies, all limited to participants of European descent to minimize population stratification bias. Our analysis was bidirectional, first assessing the causal impact of 12 micronutrients on three common respiratory infections. Summary data for these infections were obtained from two independent GWAS consortia, used for initial and replication analyses, followed by a meta-analysis for result consolidation. Secondly, we conducted subtype analyses for each respiratory infection and finally investigated reverse relationships.

Our study uses publicly accessible data from studies that already have the necessary participant consent and ethical authorization, hence our study did not require separate institutional ethics approval.

### GWAS data on micronutrients

A literature search was performed using PubMed^1^ for published GWAS data on micronutrients related to European populations, with the last search conducted on June 1, 2023. GWAS studies on cobalt, sodium, molybdenum, potassium, chromium, and vitamin K were excluded due to the absence of significant top SNPs (*p <* 5 × 10^−8^) (16–18). Initially, we identified 15 potential micronutrients: Ca (19), beta-carotene (20), Cu (21), folate (22), Fe (23), Mg (17), phosphorus (24), Se (25), vitamin A (26), vitamin B6 (27), vitamin B12 (22), vitamin C (28), vitamin D (29), vitamin E (30), and Zn (21), However, vitamins A and E were not included as their respective GWAS had been corrected for variations in body mass index (BMI), potentially biasing the genetic effects due to this adjustment (31). To avoid potential sample overlap issues between studies, we did not use GWAS data on micronutrients from the UK Biobank (32) and the FinnGen Consortium (33). Ultimately, 13 micronutrients were finalized as our exposure factors. A summary description for each exposure was provided in Supplementary Table S2.

### GWAS data on respiratory infections

Our primary MR analysis targeted three types of respiratory infections: URTI, LRTI, and pneumonia. We used GWAS data from two separate European ancestry cohorts for these outcomes, namely the UK Biobank (32) and FinnGen Release 9 (33). The UK Biobank is a substantial biomedical research database, established in 2006, containing samples from 500,000 individuals for research on diseases, genetics, and lifestyle. FinnGen is an extensive Finnish cohort study amalgamating disease endpoint genetic data from Finnish biobanks and health records. More information can be found on FinnGen’s website.^2^ We investigated the link between genetic variants in trace nutrients and respiratory infections, starting with FinnGen Release 9’s latest GWAS data as our discovery cohort, followed by replication analysis using UK Biobank data, and concluding with a combined meta-analysis to confirm our hypotheses.

Our subtype analysis involved examining summarized data from the UK Biobank and FinnGen Release 9. We identified four URTI subtypes (acute nasopharyngitis, influenza, acute pharyngitis, and other and unspecified tonsillitis), four LRTI outcomes (bronchiectasis, acute bronchiolitis, acute bronchitis, and critical care admission with LRTI), and six pneumonia disease states (asthma-related pneumonia, bacterial pneumonia, viral pneumonia, pneumonia mortality, critical care admission with pneumonia and 28-day pneumonia mortality in critical care). These were chosen as secondary outcomes to assess the connection between circulating micronutrients and the risk of different subtypes and severity levels of respiratory infections. Of the 14 subtypes analyzed, data for the different severity outcomes of LRTI and pneumonia were obtained from the Hospital Episode Statistics (HES) of the UK Biobank, with the rest sourced from FinnGen Release 9. For more details on the outcomes, see Supplementary Table S3.

### Effect size estimate and sensitivity analysis

For each micronutrient, top independent SNPs were chosen based on a rigorous threshold (*p <* 5 × 10^−8^), discarding those with linkage disequilibrium of r^2^ < 0.001 with 10,000-kb windows and palindromic SNPs. We evaluated the strength of each IV using F-statistics, with all selected SNPs presenting an F-statistic greater than 10, suggesting a low likelihood of being weak instrumental variables (Supplementary Table S4).

For MR analyses, the inverse variance-weighted (IVW) method is the main approach using a random-effects model to assess the causal impact of circulating micronutrients on respiratory infections. IVW is the main method commonly used in MR Studies, which combines all Wald ratios for each SNP to provide a summary estimate, avoiding confounding factors and obtaining an unbiased estimate of the effect size in the absence of horizontal pleiotropy (34). We also used supplementary methods, including the MR-Egger (slope-intercept) and weighted median (WM) approaches, estimating effect sizes using the Wald ratio of each SNP when only one is available. To ensure the reliability of our conclusions, sensitivity analyses were performed to examine heterogeneity and pleiotropy in the genetic factors that might skew the results of Mendelian MR. Firstly, for exposures with three or more SNPs, we checked for consistency in effect size direction across three methods (IVW, WM, and MR-Egger). Secondly, we assessed heterogeneity with Cochran’s Q test (35) across the gene IVs used in both cohorts. Thirdly, the degree of horizontal pleiotropy was evaluated using the Egger intercept method (36). Fourthly, we applied the MR-PRESSO test to identify outliers (37), and conducted the MR-PRESSO heterogeneity global test to detect the potential horizontal pleiotropy. Fifthly, leave-one-out sensitivity analysis was performed by sequentially removing each SNP, ensuring MR estimates were not driven by certain strong SNPs.

In the primary MR analysis, we conducted a replication analysis of GWAS data for URTI, LRTI, and pneumonia from the UK Biobank, assuming that both databases had complete summary statistics. We then utilized the random-effects model from the meta package (38) (version 6.5) to merge results from both the FinnGen cohorts and the UK Biobank. The combined results from this meta-analysis were regarded as the final estimates of causal effect sizes.

### Multivariate MR analysis and directivity test

To address confounding in our analysis, we performed a multivariate MR analysis. This process was aimed at identifying whether the candidate micronutrients under study independently influence respiratory infections, considering multiple genetic variations and confounding factors. We sourced summary data for two well-known risk factors associated with respiratory infections: BMI (5) and lifetime smoking (6). The BMI data were derived from the GIANT consortium (dataset IDs: ieu-a-90, ieu-a-91, ieu-a-92) (25), and the lifetime smoking data (39) were from the UK Biobank. Detailed GWAS information for these studies was provided in Supplementary Table S5.

To determine the possibility of reverse causation between the candidate micronutrients and outcomes, we undertook two directional tests. First, we performed the MR Steiger test (40) to assess the direction of the associations. Following this, a reverse MR analysis was carried out, treating respiratory infections as the exposure and the candidate micronutrients as the outcome.

### Supplementary analysis using less stringent criteria for the selection of genetic instruments

Lastly, the validation of the link between Cu and upper respiratory tract infections can be found in the results section, we performed an additional analysis. Due to the limited number of available IVs for Cu (only two) found at stricter thresholds (*p <* 5 × 10^−8^ and r^2^ < 0.001 within 10,000-kb windows), which hindered the heterogeneity and horizontal pleiotropy tests, we adopted a looser threshold (*p <* 5 × 10^−6^ and r^2^ < 0.001 within 10,000-kb windows). We then reanalyzed URTI outcomes from both FinnGen and UK Biobank cohorts using MR analysis and subsequently further compared the meta-combined MR results with the initial effects.

### Statistical analyses

All data analyses were carried out using the R package “TwoSampleMR” and “MRPRESSO” of R software 4.2.3.^3^ The GWAS meta-analyses were mainly performed using a random-effects model provided by the “meta” package (version 6.5). Considering multiple tests, the Bonferroni-adjusted level of statistical significance (for 12 exposures and 3 outcomes) was established at *p* = 1.39 × 10^−3^ (0.05/36). *p*-values falling between 0.05 and 0.00139 were deemed to have nominal significance. Power analysis was conducted using the online resource at http://cnsgenomics.com/shiny/mRnd/, where the primary parameters included the outcome’s sample size, case proportion, OR, and *R*^2^. In our research, we considered only those micronutrients with an *R*^2^ greater than 1% or those with over 50% statistical power for at least one respiratory infection outcome, thereby excluding vitamin B12 (Supplementary Tables S2, S6).

## Results

The circulating micronutrients had between 1 to 11 instrumental variables, with a median count of 4. These SNPs had F-statistics ranging from 16 to 711, with the median at 49, clearly above the traditional threshold of 10, suggesting a very low probability of weak instrument variables. One SNP for vitamin C (rs13028225) was unavailable in all outcome datasets. Two SNPs for phosphorus (rs1697421, rs9469578) were removed due to incompatible alleles.

### Impact of micronutrient levels on overall risk of respiratory infections

Following our initial selection of instruments, we conducted a preliminary evaluation of the relationship between 12 circulating micronutrients and the risks of URTI, LRTI, and pneumonia. In the FinnGen discovery cohort, we observed one significant causal relationship and two suggestive associations (Supplementary Tables S7–S9). Specifically, genetically predicted Cu levels showed a notable protective effect against the occurrence of URTI [odds ratio (OR) = 0.924, 95%CI: 0.888 to 0.963, *p =* 0.000159, IVW] (Supplementary Table S7). Sensitivity analysis results were presented in Supplementary Table S10, the *p*-value obtained from Cochran’s Q test indicated no heterogeneity (*p =* 0.419). However, given the restricted number of available SNPs, with only two available, it was not possible to conduct MR-PRESSO and MR-Egger regression analyses. Additionally, two micronutrients show suggestive associations with the outcomes: Cu (reduces the risk of URTI, *p =* 0.038, IVW) (Supplementary Table S7) and zinc (decreases the risk of pneumonia, *p =* 0.047, IVW) (Supplementary Table S9). In the sensitivity analyses of these two suggestive associations, the *p*-values from Cochran’s Q tests indicated no heterogeneity. The MR-Egger assessment for horizontal pleiotropy between the IVs and outcomes showed insufficient evidence of horizontal pleiotropy (Supplementary Table S10).

**Figure 2:**
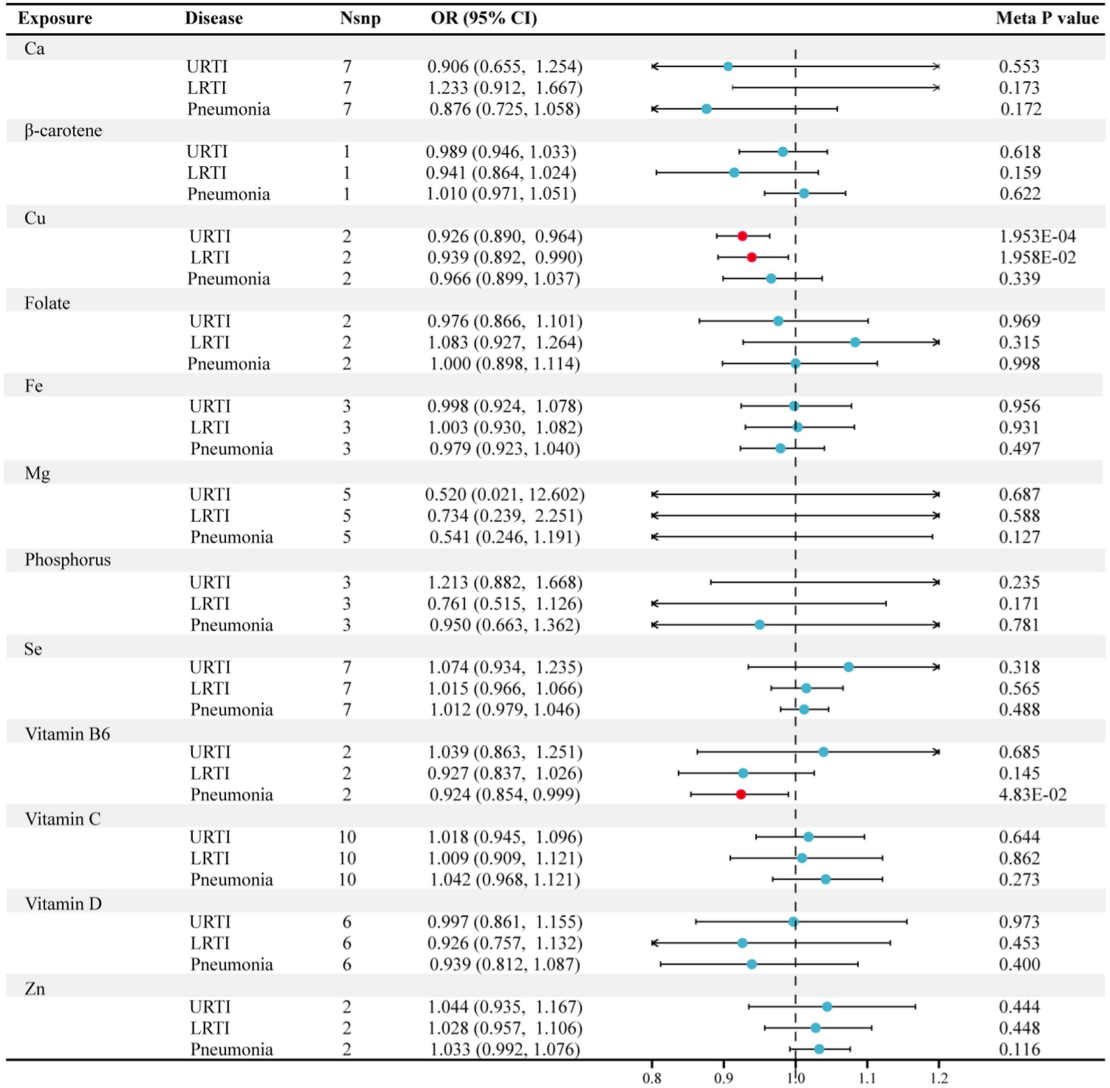
Forest plot for the meta-analysis of circulating micronutrients levels on the risk of respiratory infection. Nsnp, number of SNP; OR, odds ratio; CI, confidence interval; Ca, calcium; Cu, copper; Fe, iron, Mg, magnesium; Se, selenium; Zn, zinc.

After replication and meta-analysis in the UK Biobank, with Bonferroni correction applied, the only statistically significant association identified between micronutrients and infections was the genetically estimated levels of Cu in the blood and their association with URTI. An increase of one standard deviation (SD) in the genetically predicted blood levels of Cu was associated with an OR of 0.926 (95% CI, 0.890 to 0.964, *p =* 0.000195), as confirmed in the meta-analysis (Figure 2; Supplementary Table S7). Additionally, as shown in Supplementary Table S6, the analysis power for the causal relationship between Cu and URTI was 98%, further confirming the reliability of our results. Furthermore, the meta-analysis indicated two suggestive associations: Cu shows a nominal relationship with decreased risk of LRTI (OR = 0.939, 95% CI: 0.892 to 0.990, *p =* 0.0196) (Figure 2; Supplementary Table S8), and Vitamin B6 displays a nominal protective effect against the risk of pneumonia (OR = 0.924, 95% CI: 0.854 to 0.999, *p =* 0.048) (Figure 2; Supplementary Table S9).

We observed almost no evidence of an association between the blood levels of calcium, β-carotene, folate, iron, magnesium, phosphorus, selenium, vitamin C, vitamin D, and zinc with the overall risk of URTI, LRTI, and pneumonia (Figure 2; Supplementary Tables S7–S9).

### Associations between micronutrients and specific subtypes of respiratory infections

**Figure 3:**
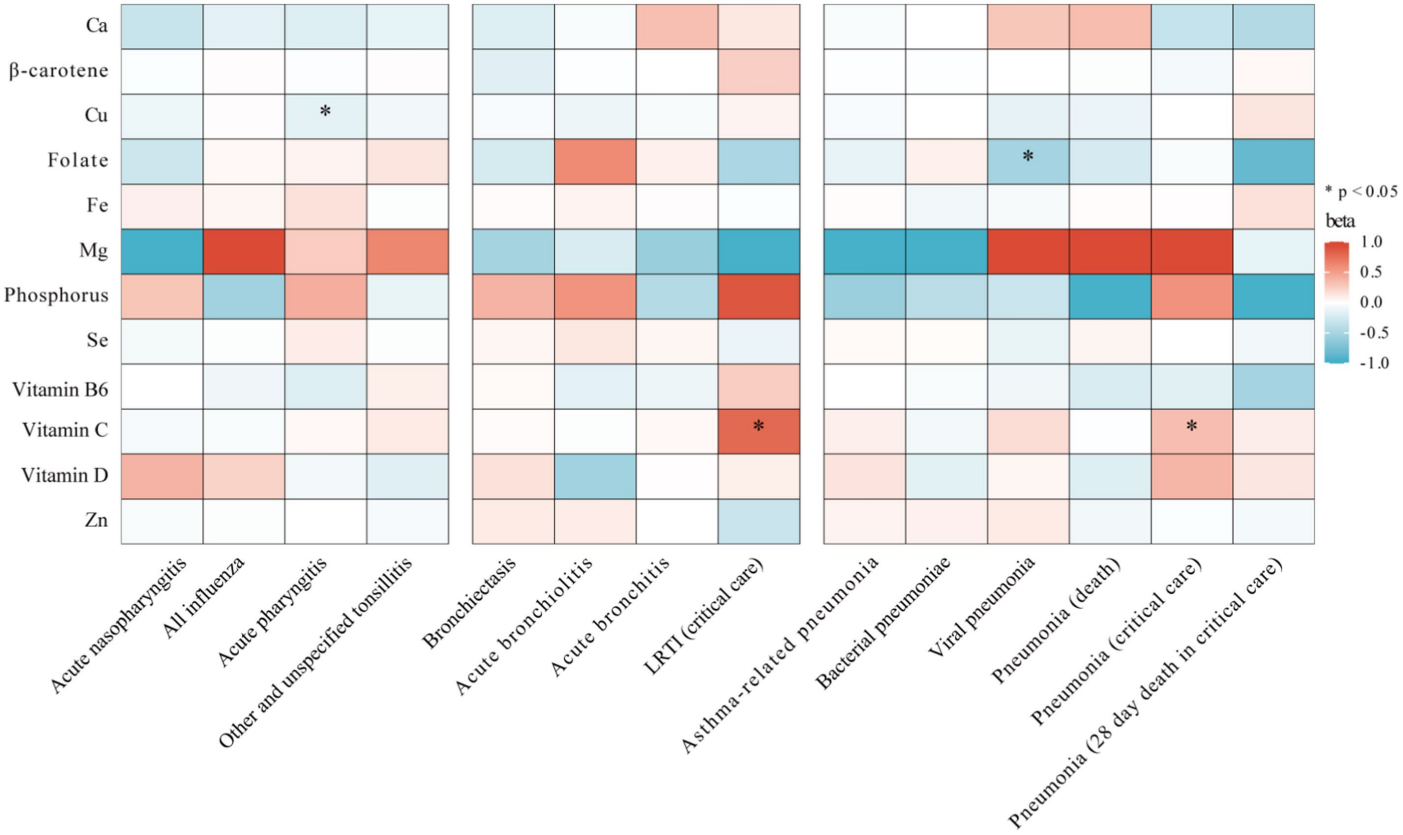
Heatmap showing the causal effects of circulating micronutrients levels on respiratory infection subtypes risk by using IVW or wald ratio method. IVW, inverse-variance weighted; Ca, calcium; Cu, copper; Fe, iron, Mg, magnesium; Se, selenium; Zn, zinc; LRTI, lower respiratory tract infections.

Our study evaluated the link between 12 micronutrients and 14 subtypes of respiratory infections, detailed in Figure 3 and Supplementary Table S11. We found four preliminary associations (*p <* 0.05) involving three micronutrients and four infection subtypes. Specifically, there appeared to be suggestive causal relationships between genetically predicted serum Cu levels and acute pharyngitis (OR = 0.855, 95% CI: 0.744 to 0.984, *p =* 0.029, IVW), vitamin C and critical care admission with LRTI (OR = 2.213, 95% CI: 1.129 to 4.338, *p =* 0.021, IVW), vitamin C and critical care admission with pneumonia (OR = 1.392, 95% CI: 1.030 to 1.882, *p =* 0.032, IVW), and folate and viral pneumonia (OR = 0.602, 95% CI: 0.368 to 0.982, *p =* 0.042, IVW). Despite this, sensitivity analyses found no evidence of heterogeneity or pleiotropy (Supplementary Table S11), and these associations did not meet our stringent statistical threshold after the Bonferroni correction.

Furthermore, no clear evidence was found linking blood levels of calcium, β-carotene, iron, magnesium, phosphorus, selenium, vitamin B6, vitamin D, or zinc with the risk of developing any of the four subtypes of URTI, four subtypes of LRTI, or six outcomes of pneumonia.

### Multivariable MR analyses and directionality test

Further multivariable MR studies assessing the influence of potential risk factors [BMI (5) and lifetime smoking (6)] on the relationship between Cu and URTI. The results, adjusted for each risk factor, indicated a similar effect as our primary analysis (Table 1). In our directionality test, the initial Steiger test did not support a reverse causal relationship between Cu and URTI (Supplementary Table S12). Furthermore, reverse MR analysis using significant, independent SNPs associated with URTI outcomes confirmed that URTI does not influence circulating Cu levels in the blood, suggesting a minimal likelihood of a reverse causal effect, as detailed in Supplementary Table S13.

**Table 1:** Multivariable Mendelian randomization analyses of Cu with common risk factors for URTI adjusting for potential risk of factors.

Outcome	Model	OR (95%CI)	*p*-value
URTI	Unadjusted model	0.926 (0.890, 0.964)	1.95E-04
	Adjusted for Obesity (Class 1)	0.920 (0.871, 0.971)	2.54E-03
	Adjusted for Obesity (Class 2)	0.930 (0.875, 0.988)	0.019
	Adjusted for Obesity (Class 3)	0.925 (0.902, 0.948)	4.25E-10
	Adjusted for Obesity (Class 1, 2 and 3)	0.921 (0.866, 0.981)	1.02E-02
	Adjusted for Lifetime smoking	0.951 (0.914, 0.988)	0.011

URTI, upper respiratory infection; OR, odds ratio; CI, confidence interval.

### Supplementary analyses

In our additional analysis exploring the link between Cu and upper respiratory infections, we used a relaxed threshold criteria (*p <* 5 × 10^−6^ and r^2^ < 0.001 over 10,000-kb windows), identifying six instrumental variables. Supplementary Table S14 shows detailed information about these six instrumental variables, with the lowest F-statistic being 21, indicating a low likelihood of weak instruments. The IVW method resulted in an OR of 1.04 (95% CI, 0.969 to 1.040, *p =* 0.842), but with notable heterogeneity (Cochran’s Q test *p =* 5.18 × 10^−3^). This heterogeneity disappeared after removing rs12582659 and rs10014072. Subsequently, utilizing the remaining four SNPs as IVs for Cu, we reconducted MR analyses on URTI in the FinnGen and UK Biobank cohorts. This meta-analysis of these results produced an effect similar to our primary findings, with an OR of 0.947 (95% CI, 0.901 to 0.995, *p =* 0.033, IVW) (Supplementary Table S15; Supplementary Figure S2).

## Discussion

This MR research focused on examining the possible connections between 12 circulating micronutrients and respiratory infections. Our findings revealed associations of potential statistical significance with five micronutrients. After multiple correction tests, we established a strong and robust causal link between one micronutrient and respiratory infections. We ruled out the possibility of reverse causation, confirming that the identified micronutrient is a precursor to infection, not a result. Specifically, high levels of genetically predicted circulating Cu in the blood were closely associated with a lower risk of upper respiratory tract infections. This research is the first of its kind to use MR methods to thoroughly examine the causal connections between various micronutrients in the blood and the susceptibility, severity, and subtypes of respiratory infections.

Cu, a key micronutrient, is instrumental in the functioning and maintenance of various immune cells, including Th cells, B cells, neutrophils, NK cells, and macrophages. Previously, there was no clear link between circulating Cu levels in the blood and the risk of human respiratory infections. An RCT revealed that supplementing burn patients with elevated levels of Cu notably decreased their infection risk (41). Another trial demonstrated that the enhancement of Cu supplementation in healthy individuals with Cu levels that were below to within the normal range led to increased interleukin-2 production from blood cells (42), which is essential for natural killer cell cytotoxicity and T-helper cell proliferation. Moreover, recent research has shown that supplementing Cu and zinc may have a positive effect on platelet activation in patients with pulmonary embolism during SARS-CoV-2 infection (43), leading to the proposal of Cu as a potential therapy during COVID-19. This aligns with our findings that high levels of Cu may offer protection against infectious diseases. A previous cellular experiment revealed a possible mechanism, showing that Cu-enriched cell cultures enhanced macrophage phagocytosis and bactericidal capabilities (44). Polarized macrophages produce more anti-inflammatory factors, such as IL-10 and TGF-β, and mitigate inflammation by inhibiting effector T cells (45). Our MR study provides genetic evidence supporting the protective effect of Cu against URTI. Additionally, we observed a suggestive association between Cu levels and a reduced risk of LRTI and acute pharyngitis, although these findings did not meet our stringent statistical criteria. These results suggest that Cu may play a beneficial role in respiratory infections and could be a promising candidate for the prevention and treatment of these conditions. Based on these findings, incorporating assessments of Cu levels into routine health screenings and exploring Cu-based nutritional interventions as part of personalized medical strategies may be beneficial for optimizing the prevention and management of infections.

We also found a suggestive negative correlation between genetically estimated blood vitamin B6 levels and the risk of pneumonia. Although previous research on Vitamin B6 and respiratory infections was sparse, its protective role against diseases was indirectly supported by extensive studies. In previous observational studies, vitamin B6 deficiency has been associated with a variety of autoimmune diseases, including rheumatoid arthritis, multiple sclerosis (46), and type I diabetes (47). Vitamin B complex can treat neuroinflammation after peripheral nerve injury (48), and multiple animal studies may explain the mechanism of this occurrence. Mice deprived of the Vitamin B6 diet showed decreased IL-2 levels and increased IL-4 levels (49). Similarly, decreases in anti-inflammatory cytokines (TGF-β, interleukin-4, interleukin-10, interleukin-11, and interleukin-13) and increases in pro-inflammatory cytokines (interleukin-1, interleukin-6, interleukin-8, interleukin-12, interleukin-15, and interleukin-17) were also found in grass carp deficient in vitamin B6, Activated the NF-κB signaling pathway (50). A recent MR Study also found a suggestive association of increased vitamin B6 levels with reduced risk of ischemic stroke (51). Unfortunately, this finding, like ours, did not pass a strict statistical threshold. In this study, we observed a suggestive association between vitamin B6 levels and reduced risk of pneumonia, indicating its potential application in the prevention and treatment of pneumonia. Larger studies are needed to further confirm our conclusions.

In addition, we observed a suggestive association between genetically predicted increases in folate levels and a reduced risk of viral pneumonia, similar to previous observational studies in which Jacobson found significantly reduced serum folate in patients with viral and *mycoplasma pneumoniae* infections (52). Sato et al.’s study also proved that folate deficiency may be an independent marker of increased risk of aspiration pneumonia in the elderly (53). Recently, Najafipou’s study also proposed that hypomethylation of ACE-2 gene caused by folate deficiency is an independent risk factor for severe acute respiratory distress syndrome (ARDS) (54). However, our MR Did not find an association between folate and pneumonia, bacterial pneumonia, asthma-related pneumonia, and the severity of pneumonia, and further studies are needed to explore the protective effect of folate on the risk of viral pneumonia.

Concerning vitamin C, earlier observational studies have suggested that vitamin C may reduce the severity and mortality risk in elderly patients with pneumonia (55). However, a recent MR study found no link between circulating vitamin C levels and the risk of developing pneumonia (56). Although our research indicated a nominal positive correlation between circulating vitamin C levels and the incidence of severe lower respiratory tract infections and severe pneumonia, this finding could be a result of multiple testing and did not meet our strict statistical significance threshold.

Interestingly, our research did not establish a causal relationship between the genetically predicted levels of circulating nutrients such as calcium, beta-carotene, vitamin D, iron, magnesium, phosphorus, selenium, and zinc, and the risk of respiratory infections, including both upper and lower tract infections and pneumonia. Some systematic reviews indicate that the effect of micronutrient supplements on infections is minimal. For example, zinc supplementation shows no impact on LRTI in infants (57), and there was no evidence supporting selenium’s role in infection prevention (58). This points to a lack of extensive research in this area. While previous MR studies have found a connection between iron and sepsis (59), we did not find any evidence linking these nutrients to the infections we studied. Earlier MR studies suggested a link between low vitamin D levels in the blood and a higher risk of pneumonia (60), but meta-analyses of vitamin D supplementation experiments contradict this idea (61). These studies may suggest that these micronutrients might not be causative factors in respiratory infections and related outcomes.

There were several strengths in our study. It stands as the inaugural MR study that thoroughly examines the association between 12 different micronutrients and the risk of respiratory infection susceptibility and severity, thus reducing potential confounders present in observational studies. Furthermore, Existing large-scale GWAS data were predominantly available for European ancestry populations, enabling us to access sufficient sample sizes for robust analyses, European ancestry populations tend to have higher genetic homogeneity, reducing heterogeneity in gene–environment interactions. Moreover, we used data from two separate GWAS databases for our three primary outcome analyses (URTI, LRTI, and pneumonia), and performed a meta-merger of these results. This approach lessens the impact of data source discrepancies and bolsters our associative analysis capabilities. Additionally, we conducted extensive sensitivity analyses to confirm the reliability of our findings.

Additionally, our research was subject to certain limitations. First, our MR approach used published aggregated data, which may not account for possible non-linear relationships. Second, our sample was limited to individuals with European ancestry, which restricts wider applicability, though it reduces bias from population stratification. Future research should aim to replicate our findings in diverse populations to ascertain the universality of the associations. Third, Subtle differences in the definition of respiratory infection subtypes across different countries and regions might affect the generalizability and accuracy of the study findings. However, by utilizing outcome data from two distinct databases, we have enhanced the consistency of the results and mitigated the impact of this heterogeneity to some extent. Fourth, although we found no associations between particular micronutrients and respiratory infection risk, we cannot completely discount the possibility that the effects of calcium, beta-carotene, iron, zinc, magnesium, phosphorus, selenium, and vitamin D were too subtle to determine a causal relationship. Therefore, future large-scale genome-wide studies were necessary to further explore the potential impact of micronutrients on infections. Finally, for Cu, our main MR analysis included only two instrumental variables, but additional analyses with relaxed thresholds and more genetic tools yielded results similar to our main analysis. This consistency was further supported by various sensitivity analyses, multivariable MR, and reverse MR, validating our key findings.

## Conclusion

Our study suggests that genetically predicted levels of Cu may be causally linked to a reduced risk of URTI. This finding offers new evidence for the prevention and treatment of respiratory infections.

## Data availability statement

The original contributions presented in the study are included in the article/Supplementary material, further inquiries can be directed to the corresponding author.

## Ethics statement

Ethics approval and consent to participate original GWAS research’s ethics approval and participant permission were available, and only publicly accessible GWAS data were used in this analysis.

## Author contributions

ZWei: Conceptualization, Data curation, Formal analysis, Investigation, Methodology, Project administration, Writing – original draft, Writing – review & editing. QX: Data curation, Validation, Writing – review & editing. LL: Data curation, Validation, Writing – review & editing. ZWu: Project administration, Writing – review & editing. ZC: Project administration, Writing – review & editing.

## Footnotes

^1^ https://www.ncbi.nlm.nih.gov/pubmed

^2^ https://finngen.gitbook.io/documentation/

^3^ https://www.r-project.org/

## Supplementary material

SUPPLEMENTARY FIGURE S1 Comprehensive flowchart of study design and analytical procedures. Abbreviations: URTI, upper respiratory infection; LRTI, lower respiratory tract infections; UVMR, Univariate Mendelian randomization; MVMR, Multivariate Mendelian randomization; BMI, body mass index.

SUPPLEMENTARY FIGURE S2 Scatter plot of MR analysis of Cu as protective factors on the risk of URTI. Abbreviations: URTI, upper respiratory infection; Cu, copper.
