# Supplementary figures and images for: Circulating micronutrient levels and respiratory infection susceptibility and severity: a bidirectional Mendelian randomization analysis
Source: Front Nutr. 2024 Aug 8;11:1373179. doi: 10.3389/fnut.2024.1373179 (PMC11338864; doi:10.3389/fnut.2024.1373179)

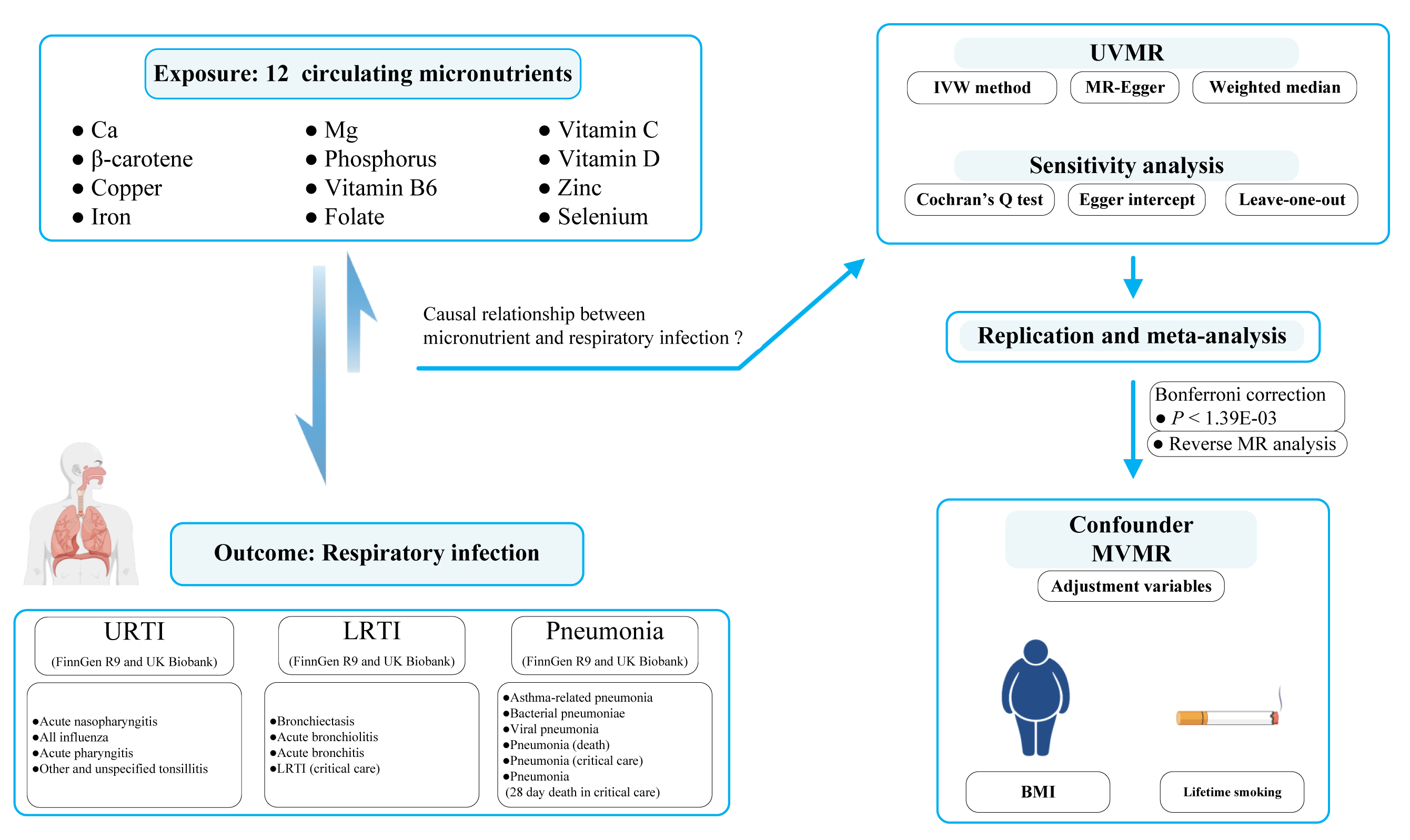

Supplement: Supplementary file 1 [file Data_Sheet_1.zip › Supplementary Figure S1.tif]

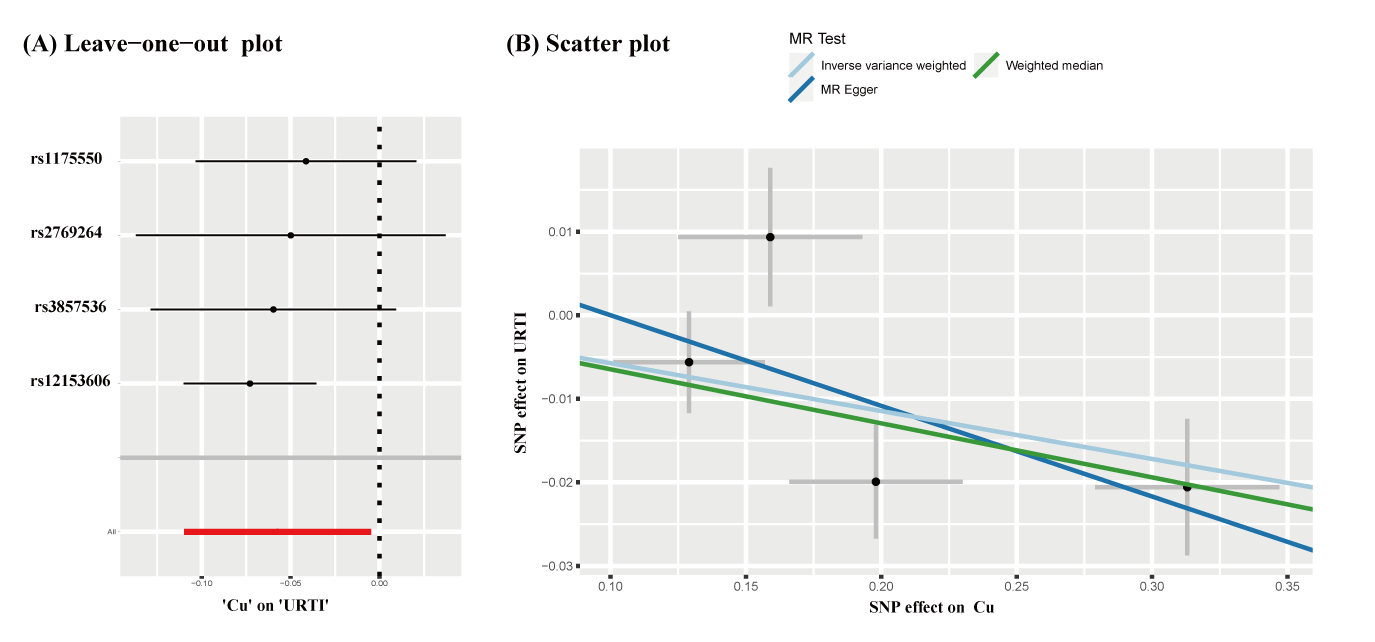

Supplement: Supplementary file 1 [file Data_Sheet_1.zip › Supplementary Figure S2.tif]
